# Fibre Optic Sensors for Selected Wastewater Characteristics

**DOI:** 10.3390/s130708640

**Published:** 2013-07-05

**Authors:** Su Sin Chong, A. R. Abdul Aziz, Sulaiman W. Harun

**Affiliations:** 1 Department of Chemical Engineering, University of Malaya, 50603 Kuala Lumpur, Malaysia; E-Mail: susin_2117@yahoo.com; 2 Department of Electrical Engineering, University of Malaya, 50603 Kuala Lumpur, Malaysia; E-Mail: swharun@um.edu.my

**Keywords:** online monitoring, fibre optic, chemical oxygen demand, biological oxygen demand, colour, optical sensor, environmental sensing

## Abstract

Demand for online and real-time measurements techniques to meet environmental regulation and treatment compliance are increasing. However the conventional techniques, which involve scheduled sampling and chemical analysis can be expensive and time consuming. Therefore cheaper and faster alternatives to monitor wastewater characteristics are required as alternatives to conventional methods. This paper reviews existing conventional techniques and optical and fibre optic sensors to determine selected wastewater characteristics which are colour, Chemical Oxygen Demand (COD) and Biological Oxygen Demand (BOD). The review confirms that with appropriate configuration, calibration and fibre features the parameters can be determined with accuracy comparable to conventional method. With more research in this area, the potential for using FOS for online and real-time measurement of more wastewater parameters for various types of industrial effluent are promising.

## 1. Introduction

One of the challenges of industrialization is the effects of wastes by-products on the environment, of which wastewater from industrial activities is a major contributors to water pollution in Malaysia due to the copious amount of chemicals used in the processes. According to a Malaysian Department of Environmental (DOE) report, the amount of industrial effluents from various municipalities had inevitably increased from 1,103,457.06 metric tons in 2006 to1,622,031 metric tons in 2011 [1,2]. Such wastewaters are abundant with chemical residue containing heavy metal and colouring.

Wastewater effluent quality can change rapidly in the event of treatment plant performance failure. Unconstrained continuous water quality monitoring is difficult to achieve due to limited space within the sewer system and its separation from the laboratory [3]. In order to mitigate the effects of the compromised environment and comply with stringent government legislation, close performance monitoring of each of the treatment plant needs to be done on a regular basis. Spectroscopic techniques have been acknowledged as the most compelling spectrum analysis techniques since the 1990s. They have the advantages of being relatively inexpensive, having short test times and not requiring reagents or sample preparation. In addition, their ability to measure directly makes them suitable for on-site determination. A breakthrough in optical fibre spectroscopy research from Kao and Hockham [4] reveled that light which is guided through a thin flexible dielectric fibre has low loss in its waveguides. Technology has since taken optical fibre sensors to new heights of innovation and proffered an outstanding solution to certain challenges [5]. There have been more studies on the accuracy, reliability, portability and cost effectiveness of optical fibre sensors, and it can be become a competitive alternative to the conventional sensors. A previous experiment has reported that optical fibre spectroscopy is a well-suited technique for online monitoring in food quality and safety assessment [6]. When combined with multivariate calibration methods, higher accuracy and precision can be achieved for monitoring purposes, especially of wastewater treatment plant [7].

## 2. Conventional Detection Method for Parameters of Colour, COD, and BOD

Conventional methods of colour, COD and BOD detection in a laboratory (off-line) are often applied to measure the parameters that measures pollutant levels in industrial wastewater [8,9]. These methods have been summarized in Table 1. However, results obtained through these methods only provide snapshots of moments in time, which are insufficient for sampling the highly changeable quality of effluent. Rapid, continuous monitoring in real time and record keeping by the industry has to be prioritized.

### 2.1. Colour

Colour in wastewater is normally caused by dissolved matters after the removal of turbidity [9]. The majority of industrial wastes are high in colour intensity, especially those of the textile industries. Dyes contained in wastewater that are not completely removed by treatment and are later discharged into drains are potentially carcinogenic due to their benzidine content and high compositional variability [21]. Off-line monitoring test (sample handling needed) is not suitable for use in this kind of monitoring unit operation at the wastewater treatment plant. The concentration of dyestuffs found in the environment can cause environmental risks due to its inherent eco-toxicology. Thus, reliable analytical methods are needed to detect critical concentrations of dye compounds in the environmental compartment [22].

### 2.2. Chemical Oxygen Demand

Chemical Oxygen Demand (COD) is used as a measure of oxygen required to oxidize organic matter of a sample by a strong chemical oxidant. Potassium dichromate, for instance has been widely used for oxidizing a variety of organic substances completely. When the reaction is complete, colourimetric quantification of the residual oxidant is performed [9]. The residue becomes an important parameter and it is used extensively to determine the quality of wastewater quickly (around 2 h), compared with 5 days for a Biological Oxygen Demand test. A number of improvements have been carried out on the digestion process as shown in Table 1. However, most industrial wastewater sample contains chloride ions, aromatic hydrocarbons and pyridine, which are not oxidized unless a catalyst like silver sulphate (Ag_2_SO_4_) of used [23]. Consequently, secondary hazardous waste is produced and need special disposal.

### 2.3. Biochemical Oxygen Demand

Biochemical Oxygen Demand (BOD) is one of the widely used parameters to determine the relative oxygen requirements to oxidize the organic matter which present in industrial effluent when seeded with a microbial system [9]. A quantitative relationship for oxygen demand of microorganism to oxidized wastewater sample to carbon dioxide, water and ammonia is shown as an equation [23] below:

(1) $${\text{C}}_{\text{n}}{\text{H}}_{\text{a}}{\text{O}}_{\text{b}}{\text{N}}_{\text{c}}+\left(\text{n}+\frac{\text{a}}{4}‐\frac{\text{b}}{2}‐\frac{3}{4}\text{c}\right){\text{O}}_{2}\to {\text{nCO}}_{2}+\left(\frac{\text{a}}{2}‐\frac{3}{2}\text{c}\right){\text{H}}_{2}\text{O}+{\text{cNH}}_{3}$$

Variations of oxygen demand requirement exist, which include using shorter and longer incubation duration, when using respirometric method for measurement of oxygen uptake (units mg O_2_/L) [9]. There are a few methods for the determination of BOD, including microbial sensor demonstrated by Riedel *et al.* [17]. The authorized method for BOD adopted by the American Public Health Association Standard Methods Committee [9] requires time and the use of careful laboratory quality control practices to complete the test, in which cumbersome procedures are involved. As such, it is inadequate for on-line monitoring wastewater treatment unit operations.

## 3. Online Measurements

Continuous water quality monitoring at the plant does not just provide hydrological data; it also provides a true picture and real time recognition of changes in order to alert the plant operators if the quality of effluents matches up with the established criteria. The alternative methods of colour, COD and BOD detection which are introduced in this review are based on optical working principles, for instance, optical sensors and optical fibre sensors.

### 3.1. Optical Sensor

Dissolved organic compound in wastewater contains aromatic structures, which have absorption peaks within the UV region [24,25]. Due to these characteristics, some linear relationships are established within them, which include water total organic carbon (TOC), nitrate, suspended solids (SS), COD, BOD and dissolved organic carbon (DOC). The extent of water pollution can be judged directly via the absorbance value after a water sample is irradiated with 254 nm UV light [26]. According to research [27,28], the UV light absorbance at 254 nm has been utilized to directly estimate the aggregate organic content of a water sample. Rapid and versatile monitoring of colour, BOD and COD using optical techniques such as UV-visible spectroscopy and fluorescence measurements was suggested in the research of Bourgeois *et al.* [29]. Optical properties such as absorption, scattering, re-emission and reflection can be used to rapidly identify the characteristic of wastewater, even in large batches of samples [25].

### 3.2. Optical Fibre Sensor

Essentially, fibre optic sensors (FOS) work like other electrical sensors except that the FOS uses a glass fibre instead of copper wire and light instead of electricity. A FOS modulates the properties of light including intensity, phase, polarization, or wavelength shift [30]. To date, much of the instrumentation improvements in this particular application have been recorded. Different sensor applications require different types of transducers (optical fibres) to manipulate the incident radiation. Thus, instrumentation for optical fibre sensor requires an overview.

#### Fibre Sensor System Configuration

**Figure 1.**
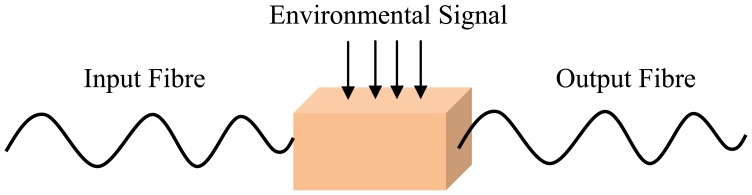
Extrinsic FOS (adapted from [31]).

**Figure 2.**
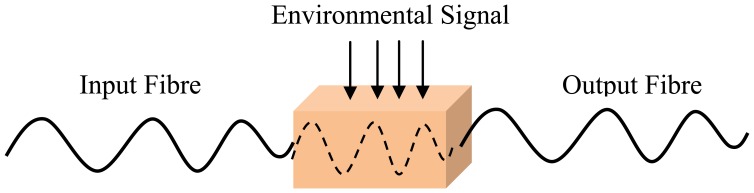
Intrinsic FOS (adapted from [31]).

An optical fibre sensing system is basically composed of a light source, optical fibre, a sensing element or transducer, and a detector. Brief descriptions of FOS components are given in Table 2. The basic optical fibre structure consists of a core surrounded by some cladding material of a lower refractive index and both are transparent dielectric cylindrical. The requirement for FOS is to have a low optical and mechanical attenuation under all anticipated operational conditions without performance degradation [5]. FOS can be categorized into two groups: intrinsic and extrinsic [31]. Figures 1 and 2 show a basic construction of extrinsic FOS and intrinsic FOS, respectively. So-called intrinsic devices rely on a light beam propagating through the fibre and interaction occurs within the environmental effect and the optical fibre itself. Extrinsic fibre optic devices are where the optical fibre is used to couple light. The light beam transmits and passes out of fibre to the exposed environment effect. Then the light doubles back to the fibre again. Consequently, the light beam to and from the fibre region are influenced by the measurand [32].

**Figure 3.**
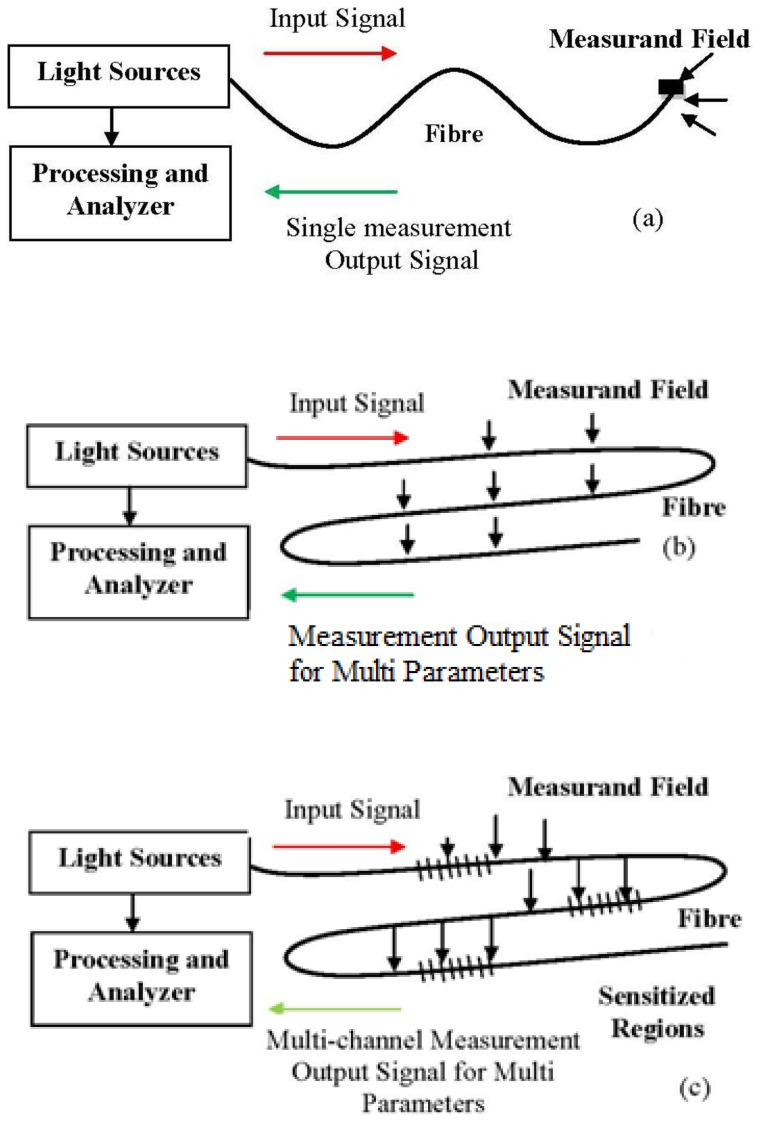
Basic fibre sensor system configuration (adapted from [32]).

Optical fibres take advantage of their unique characteristics, which are susceptibility to linear and non-linear environmental effects to enable distributed sensing. So, to achieve optimum performance to a given measurand, a detailed system design which matches the system to the environment is necessary [32]. A majority of FOS have the propensity to operate in the way that is depicted in Figures 3(a–c). Figure 3(a) depicts a sensor tip sensitized to respond to the measurand parameter which then detects the amount of light reflected at the tip. The sensor element can provide an extended sensing response with a long length of fibre [36]. This sensitivity can be enhanced by wrapping the fibre in a compact form to act as the transducer head. Fibre optic sensing practices can offer other interesting sensing options as well, including the ability to spatially discriminate the measurand at diverse locations along an identical fibre length, as illustrated in Figure 3(b). FOS is compact and lightweight, thus allowing the possibility of having distributed or quasi-distributed sensing geometries [37]. That means FOS can be multiplexed and be capable of measurement over a continuous region or in some region (multi-channel) with a large number of discrete points of the sensing geometries, as shown in Figure 3(c). This would otherwise be cost consuming or intricate to do using conventional sensors. Using FOS, it is possible to measure many external parameters such as pressure [38], acoustics [39], strain [40], pH [41], temperature [42], heavy metal [43] with greater precision and speed.

## 4. Characteristics of FOS Development

**Figure 4.**
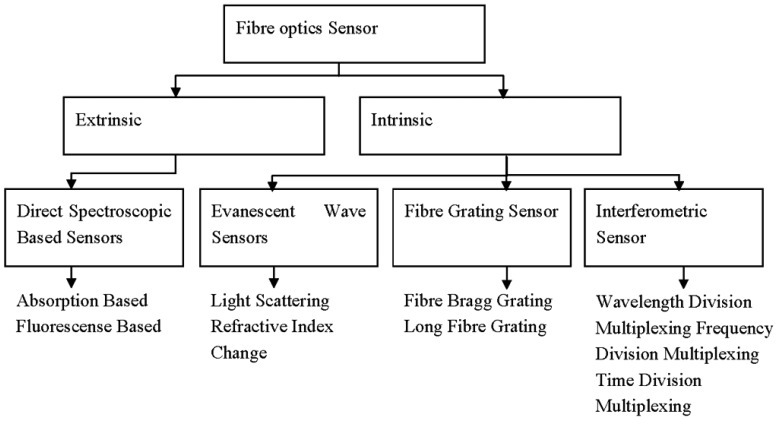
Design of fibre optics sensors.

FOS possesses several advantages over conventional devices, mainly due to the characteristics of the optical fibre itself. FOS can be made very small and thin, resistant to harsh (chemical) environments and impervious to electromagnetic interference. They can be used in remote areas that are tackling difficult measurement situations. The devices are also inherently safe because of the low optical power and the absence of electric current at the sensing point [33]. They always relate absorption and fluorescence based measurements to the target analyte [44] due to every atom, ion or molecule with its unique characteristic relationship with electromagnetic radiation. Thus, the study of optical sensors is a study of colours, concentrations, sensitivity and selectivity which leads to the analysis of the chemical species, and is specially designed to meet the requirements of many demanding applications [5]. Design of extrinsic and intrinsic sensor types with various fibre optic based on optical transduction mechanisms can be further classified as shown in Figure 4, and as discussed below. They include direct spectroscopy, evanescent wave, fibre grating and interferometric sensors.

### 4.1. Direct Spectroscopic Based Sensors

#### 4.1.1. Absorption Based

Molecular absorption spectroscopy is a common detection technique in laboratories due to its flexible adaptation to a wide variety of analytical problems [45]. Attenuation will occur when there is absorption of optical energy, which only occurs when there are a transition atoms or molecules in energetic states. However, the absorption process will only take place when the difference in the energy states involved matches with the energy of the excited photons. Specifically, the attenuation will only occur for the energy at a particular corresponding frequency. The Beer-Lambert equation shows the relationship between input and output light intensity of the incident radiation. The equation can be expressed as below:

(2) $$I={\text{I}}_{\text{o}}e^{-\alpha \text{L}}$$

where:

I_o_ is incident intensity, in unit radiant power per wavelength (W·m^−1^).
I is transmitted intensity, in unit radiant power per wavelength (W·m^−1^).
α is attenuation coefficient, in unit reciprocal length (m^−1^).
L is length of fibre, in unit metre (m).

The equation shows that there is linear relationship between intensity variation and attenuation of propagating of light. According George *et al.* [46], the strong absorption of inorganic salt compounds is located within the wavelength range from 550 nm to 750 nm. The detailed description of Nataraja *et al.* [26] about organic samples reports good absorbance in the 250 nm to 300 nm range that correlates with the concentration of the compound(s) of interest.

#### 4.1.2. Fluorescence Based

Especially in fluorescence based fibres, FOS that require short wavelength light sources (e.g., in the ultraviolet, visible or near-visible regions) are important for monitoring the chemical properties of materials. Fluorescent- based techniques are a complement to absorption based techniques for FOS [36]. In fluorescence, the electrons of the species undergoing the transition do not change their spin. The fluorescence spectrums approximately mirror the absorption spectrum. The intensity of the fluorescence spectrum depends upon the excitation wavelength. The intensity of fluorescence, F [47,48], can be readily related to the concentration of fluoresces, C, by the relationship:

(3) F=KεIC

where:

K is the quantum efficiency (unitless).
*ε* is the molar absorptivity (L/mol/cm).
I is the intensity of the incident light.
C is the concentration of fluoresces (mol/L).

Any fluorescence response is highly affected by a few factors which include temperature, concentration, pH and salinity of the solution [49]. Quenching properties can be enhanced by increasing the temperature. This study had been demonstrated by research [50], which apparently showed fluorescence intensities of dissolved organic matter increase by around 48% when the temperature increases from 10 °C to 45 °C. These results are in accordance with comparisons between the fluorophore tryptophan and fulvic acid.

There is limited information about quenching effect induced by metal ions. Keltona *et al.* set up an experiment where the metal quenching effects of dissolved organic matter from different streams were investigated. However, metals as well as irradiation diminish the capability of the optical properties to discriminate organic matter. Thus, the quenching effect also increases from 12% to 38% [51].

### 4.2. Evanescent Wave Based

A common notation evanescent wave (EW)-based method replaces a portion of the original fibre cladding with a modified cladding material (sensitive material) called taper fibre [52]. This taper fibre creates the evanescent field, resulting a light scattering phenomenon on the optical fibre structure. When radiation losses increase, the transmitted light decreases along the fibre [53]. One popular intensity modulation technique involves bending the fibre to induce radiation losses [36]. Thus, with interaction within fibre and target analyte, the optical absorption result, refractive index change or scattering information can be obtained.

**Figure 5.**
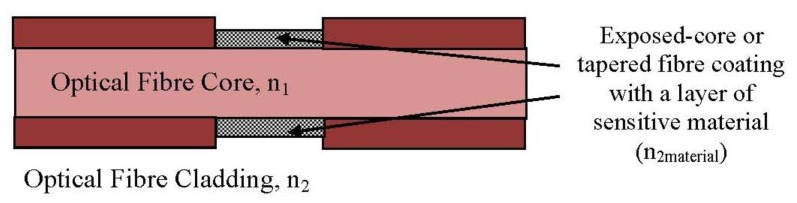
Structure of an intrinsic fibre optic sensing part (adapted from [52]).

A basic structure of an intrinsic fibre optic sensing part is shown in Figure 5. The total internal reflection (TIR) effect is observed in the resulting attenuation when the condition for critical angle criterion is achieved [54]. Interference between the incident and reflected signals at each point of TIR then generates a wave which extends beyond the optical fibre core. Light propagating along a tapered fibre is not confined to the core region but penetrates into the surrounding cladding region, the so-called penetration depth. The penetration depth can also be derived by Equation (4) [55]:

(4) $$d_{\text{p}}=\frac{\lambda }{2\pi n_{\text{core}}{\left[\sin^{2}\theta -{\left(\frac{n_{\text{clad}}}{n_{\text{core}}}\right)}^{2}\right]}^{1/2}}$$

where:

*d*_p_ is the penetration depth.
*λ* is the wavelength of the propagating signal in the optical fibre.
*θ* is the angle of incidence normal at the interface. n_core_ and n_clad_ are the refractive indices of the fibre core and cladding respectively.

#### Refractive Index

Refractive index changes are frequently demonstrated in EW sensing approaches. Any change in the optical or structural characteristic of the chemical provokes a change in the effective index of the optical fibre, thus changing its transmission properties [56]. There is great interest in these types of sensor since they offer high sensitivity, absolute detection and broad measuring ranges [57].

The refractive index of the modified cladding material can be considered in two situations: (a) having a lower refractive index than the core and (b) having a higher refractive index than the core. If the modified cladding, *n*_2_ has a lower refractive index than core *n_1_* (*n*_2_ < *n*_1_), the incident ray bends away from normal and greater than the critical angle. Then the total reflection condition is met [33]. Moreover, if the modified cladding, *n*_2_ has a higher refractive index than the core *n1* (*n*_2_ > *n*_1_), the incident ray bends towards to normal and less than the critical angle. A portion of the propagated light is refracted into the cladding, and another portion is reflected back into the core [53]. The partial leaky to mode sensor is constructed based on the intensity modulation induced by the absorption of the refracted rays and the evanescent field in the modified cladding [52].

### 4.3. Fibre Gratings

**Figure 6.**
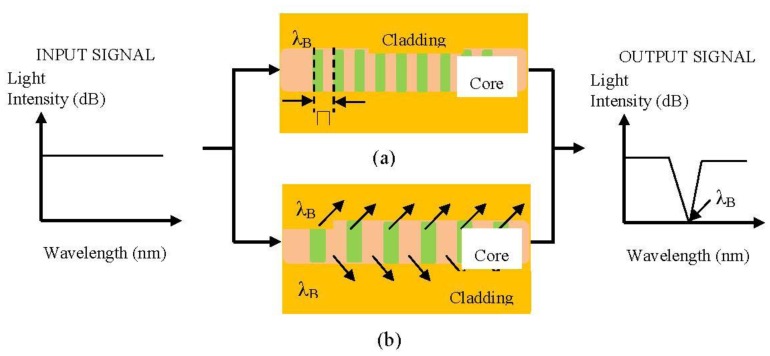
Schematic working principles with transmissive or reflective detection of (**a**) FBG and (**b**) LPG [53].

Fibre gratings have widespread application in telecommunications. Its shares common advantages with many other FOS relative to electrical sensor technology. Fibre gratings also have their own unique characteristics which include compatibility with multiplexing and distributed sensing capabilities [36]. There are two commonly used techniques associated with the fabrication of fibre gratings, namely, Fibre Bragg Grating (FBG) and Long Period Grating (LPG). A basic Bragg grating based sensor system working principle is shown in Figure 6.

FBG is known to be very susceptible to minute changes in refractive index. The fundamental principle of operation of an FBG sensor system lies in the monitoring of the wavelength shift of the reflected back Bragg signal [58]. These characteristics of fibre grating-based sensor systems are applied in conjunction with chemical sensor applications due to their ability to operate over a broad spectral range (LPG) and even individual gratings [59]. A simple formula for as grating is represented by Equation (5) [60]:

(5) $$\lambda_{\text{B}}=2.n_{\text{eff}}.\Lambda$$

where:

*λ*_B_ is the Bragg wavelength.
*n*_eff_ is the effective refractive index of the material.
*Λ* is the grating pitch (shorter than lower than the working wavelength.

### 4.4. Interferometric Techniques

The length and index of refraction of an optical fibre will change when it is subjected to an environmental perturbation. Interferometric techniques may be used to sense these changes with a high level of measurement sensitivity that matches those achieved with conventional sensor technology [61]. Various types of fibre optic implementation of the two beam interferometer sensor such as the Mach– Zehnder, Michelson; multi beam interferometer sensors such as Sagnac and Fabry–Perot, can be used to perform the detection [36]. Generally, interferometric sensors can provide a sophisticated of sensitivity to the measurement parameters if the technique used is appropriate. There are three major multiplexing arrangements characteristically used, namely Wavelength Division (WDM), Frequency Division (FDM), and Time Division (TDM) multiplexing [32].

## 5. Enhancement for FOS Sensing

**Figure 7.**
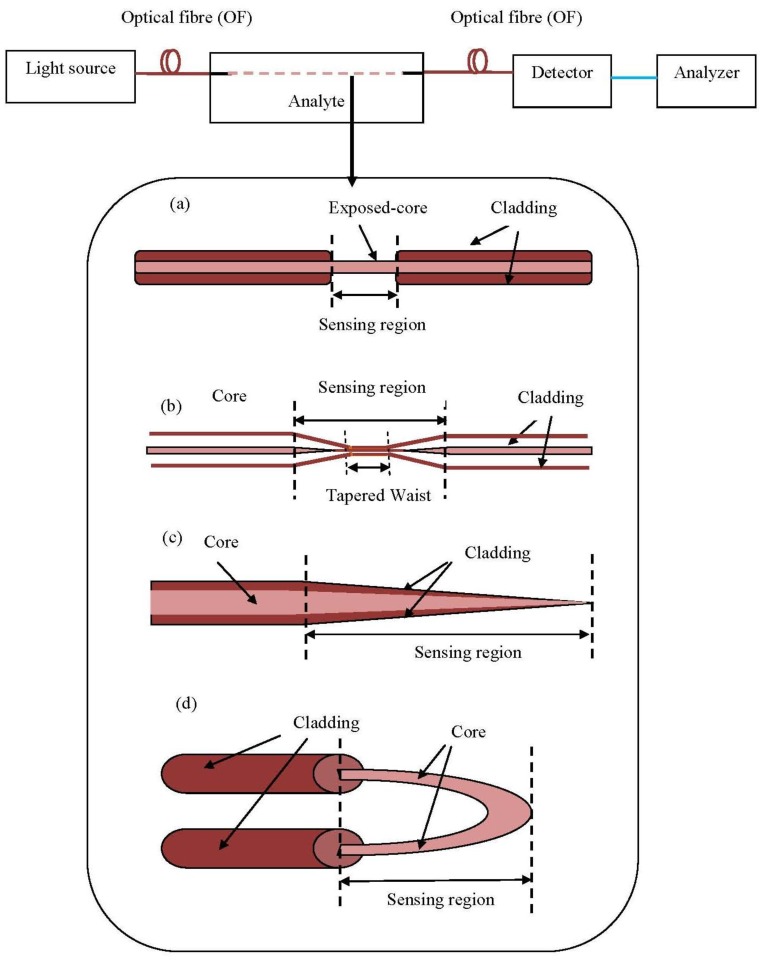
Fibre optics sensors array with few types of geometries etched fibre for sensing purpose, there are including (**a**) De-claded OF; (**b**) Tapered OF; (**c**) Tip OF and (**d**) U-bent OF (adapted from [62]).

The fibre optics industry still has high development potential. However, there is a wide variation in package designs and configurations because there are no existing standards for FOS. The choice of proper sensors and the configuration depend on the types of wastewater. A simple scheme for FOS is shown in Figure 7.

The advent of microstructure optical fibres has attracted the interest of researchers and opened up a variety of uses for photonic sensing [63]. Different portions of the optic fibre tapered for evanescent sensing purposes can be etched using chemical [64], chemical cum heat supply [65] or flame brushing [66]. Several regular fibre geometries for evanescent sensing based on optical principles are illustrated in Figure 7. Besides, U bent mode can be considered as a category of evanescent sensor too [67].

The main point of a tapered fibre is the fact that it can enhance the power fraction in the cladding, and this increases its sensitivity to environmental changes. However, after the removal of the cladding or tapered glass fibre, the device becomes very fragile and thus requires cumbersome handling procedures. As a substitution for silica fibre, plastic optical fibres (POF) have attracted increasing interest because of their robust physical and mechanical behavior. POF high break down strain is around 30% [68], whereas the silica-based fibres are fragile and will fail under a strain of 5%. The ease of use, related to simple end preparation and achieving low cost implementations, makes POF a suitable alternative material to replace silica fibres [69,70].

Besides, in order to improve on the sensitivity of FOS for chemical parameter detection, as reported in [71], no special coating is needed. Some researchers have employed a coating on a tapered or core-exposed fibre to enhance the selectivity of the device for detection. Few examples of this are found in the literature, which goes beyond the three parameters mentioned in the title; they include UV [72], ammonia vapor [73], hydrogen [63], pH [74] sensing and so on.

## 6. Loss in Optical Fibres

The attenuation, α, of an optical beam power is typically measured in unit decibels (dB). If an input power, P_1_, give rise to an output power, P_2_, then the loss in decibels, *α* is specified by [75]:

(6) $$\alpha =10\cdot \log\frac{P1}{P2}$$

The losses are due to absorption, which occur because of impurities introduced to the fibre during the fabrication process. The presence of transition metal ions could result in attenuation that would lead to losses even when present in a very small amount (1 ppm), see Table 3. Meanwhile, Table 4 shows the representative wavelength dependence of attenuation for a silica fibre. Notice that the lowest attenuation is attained at 1,550 nm. For wavelengths longer than 1,550 nm, the attenuation increases as a consequence of the absorption of infrared light by the silica molecules themselves [33,75].

An overview of the major work referenced in this review, together with method and analysis for the sensor to allow comparison and evaluation where possible are presented in Table 5.

## 7. Applications

The use of FOS is leading to the construction of reagentless methods for chemical or pollutant monitoring has rapidly gained attention. Recent reports offer some potential incorporation solutions including trace cobalt in seawater [90]; *in-situ* monitoring of aromatic hydrocarbons in contaminated groundwater was also investigated by Bureck *et al.* [91]. It is encouraging that FOS is used this way for contaminant water on-site measurement and process analysis. The following literature will focus on colour, COD and BOD detection.

### 7.1. Colour Detection

Natural and synthetic dyes are extensively used in the food processing, pharmaceutical, textile industries and so on [92]. The wastewater that they generate is a common environmental problem, especially textile waste, which contains high level of dyes. Typically, such wastewaters contain organic compounds with complex structures that are highly soluble and easily hydrolyzed [79]. Approximately 20% to 40% of these dyes remain in the effluent and some are known to be toxic and carcinogenic [93]. Indeed, colour can be characterized by using visible region light source for absorption spectrum analysis. The strong absorption band can be tailored by ring substitution. In fact, in azo groups, structural geometrical changes occur upon absorption of light [72,94]. Colour can be fully characterized by the analysis of the absorption or transmission spectrum by means of optical fibre spectroscopy [77,95].

Spectrophotometry methods with a broad wavelength range have been chosen due to the fact the colour absorption band is highly correlated with the dye concentration in the substrate. This allows the synchronized and expeditious determination of the dyes studied [77]. A single spectral value (635 nm) for monitoring the dyebath was discussed by Sahin and Ulgen [78], and was used for the continuous monitoring system of denim yarns. Thus, choosing the suitable optical source for the specified application is very important. FOS requires data processing of the output signal to extract the desired measurement from the overwhelming amount of data. First stage information based on absorption spectra shape can be used for further classification by using optical spectral analysis. For example, optical spectral analysis accomplished through mathematical methods, such as principal component analysis (PCA) using partial least squares (PLS) algorithm to analyse interferences, usually exhibit overlapped spectra in dye mixtures [77]; or optical spectral analysis using multiple linear regression model to evaluate transmission and absorption spectra of dyes, and automated correction, have been applied in this research to get highly precise values for dye samples [79].

### 7.2. COD Detection

Oxygen demand is an important index for assessing the concentration of organic matter. Concentration of organic compounds can be estimated based on the amount of oxygen used for its complete degradation. Prediction of COD due to actual COD is related to oxidizeable minerals, carbohydrates, biodegradable organic matters and humic substances [28]. Due to the characteristics of aromatic substances and double bonds that exist in their molecular structures, most organic matters have absorption peaks, particularly in the UV region and absorbance at 254–280 nm [96,97].

Wastewater contains turbidity and suspended solid (SS) which can strongly affect the transmitted optical signals [98]. Measurement corrections have to be made using different wavelengths simultaneously. Research by Matsche and Stumwohrer in 1996 [27] showed that by using two different light source wavelengths, 254 nm and 350 nm, in wastewater, biological treatment plants can reduce the effect of light absorption from turbidity and suspended solids. This important finding in ultraviolet spectroscopy for rapid determination COD was then applied in research [80,82].

An alternate approach would be the use of optical techniques to monitor the quality of wastewater. According to investigation by Dornbush and Ryckman [99], 250 nm is a useful wavelength for absorbance measurements which can be applied in analysing organic contaminants.

In addition, according to [100], oxygen demand can be evaluated by using the 280 nm wavelength as absorbance value. Studies have illustrated the high correlation between UV absorption and organic matter in wastewater, Zhao *et al.* [81] have recently demonstrated a COD monitoring system that uses the UV-Vis spectrometry absorption method. With a wide optical range of 200 to 720 nm, a wider range of COD concentration can be covered, thus, COD concentration can be calculated from 30 to 1,000 mg/L via UV absorbance within an interval of 5 min. The main role of this research is to determine the capability of monitoring COD continuously and its potential use in on-line monitoring for surface waters.

Wu *et al.* [24] then proposed a similar sensor but with the addition of near infrared (IR) transmission for comparisons to UV absorbance as a method to quantify COD. Hydrocarbons in water can be determined by absorption spectroscopy in the infrared region [101]. In the ultraviolet and visible region, particular organic contaminants can be measured quantitatively, but no qualitative information can be provided. Near infrared (NIR) spectra are capable of providing quantitative as well as some qualitative information. Meanwhile infrared (IR) spectra can grant sufficient information for a distinct identification of the analyte [102]. The NIR method is appropriate for short paths because the light scattering coefficient is larger than that of the UV method in water. NIR transmission illustrates the technical advantages of rapid, highly selective, *in situ* monitoring [103].

A fluorescence approach for COD sensing was discussed by Hur *et al.* [3]. In their work, synchronous fluorescence spectra have been studied to estimate the COD concentration of sewer samples. In order to achieve a more sensitive fluorescence-based COD sensor, Hur *et al.* [82] used a fluorescence excitation-emission matrix with PARAFAC, combined with UV absorption indices, for real time BOD, COD and Total Nitrogen concentration evaluation of urban river waters using 220 nm and 254 nm optical sources. Optical techniques can be advanced by using fibre optics. This advanced technique had been introduced by Fang and Dai [83] for predicting the COD value of waste water. It only took 62 seconds of response time and the detection range from 0 to 350 mg/L was covered.

### 7.3. BOD Detection

Contemporary study reviews are more focused on absorption-based and fluorescent-based techniques for BOD fibre sensor application [28]. The absorption of excited and emitted wavelength by the sample can result in a fluorescence intensity attenuation known as the inner filtering effect. Inner filter effects are caused by the existence of other chromophores in the solution, which absorb in the same wavelength as water samples [104]. The common solution to minimize the effect is to apply some form of mathematical correction during the calculations for the diluted samples [88], most often based on the measured absorbance of the sample [105] or fluorescence intensity multiplied by a correction factor [106].

Larsson *et al.* [107] and Ahmad *et al.* [84] suggested using Raman scatter peaks to compensate for the attenuation of the incident excitation radiation. Henderson *et al.* [104] utilized the relationship between concentration and fluorescent intensity to relate a non-linear effect with wavelength and left the data uncorrected. Special attention and correction should be paid towards the fluorescence spectra, even though altering the pH of the original sample is not recommended [108]. As observed in the research by Reynolds *et al.* [109], fluorescence intensities increase with pH until 10 for raw sewage samples. According to the research of Patel-Sorentino *et al.* [110], similar results were obtained for fluorescence intensity, which increased to 10 due to the structure of humic substances that changed with the modifications in their environment. Most problems arise at highly concentrations of contaminant samples, in such case, filtration, dilution and absorption tests out for inner filter effect are suggested.

Despite this usefulness as a monitoring tool, however, fluorescence studies have simply relied on “sing e peak i king” to quantify the desired fluorescent components. Synchronous fluorescence can be used by fixing the excitation and emission wavelengths uniformly to get the spectra from a scan of the entire section of the excitation wavelength. This is an advanced way to obtain a better resolution of fluorescence spectra. This is because it can capture all fluorophores contained in the samples [111].

**Figure 8.**
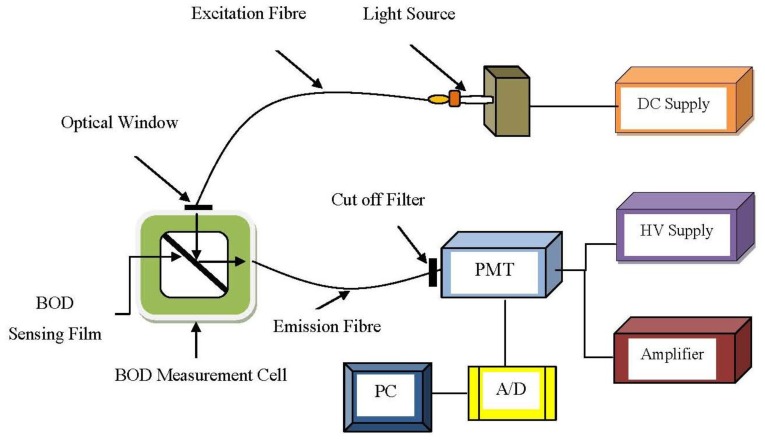
Optical fibre system for BOD measurement (adapted from [89]).

Fluorescence-based techniques with biosensors can be dividing into two general types: sandwich assays and direct methods. For the sandwich assay method, a primary immobilized cell is bonded to the surface of the fibre. The presence of target bio-molecule that bind (fluoresce) with the immobilized cell can then be excited by incident light and emission fluorescent [62]. Relations between fluorescence properties and natural waters were reported as early as 1910 by Dienart [112]. Preliminary results on the fluorescence and scattering properties of sewage have been established as well in 1993 [113]. In addition, the total integrated fluorescence intensity contributed by suspended material, the dissolved biodegradable or non-biodegradable organic matter can be correlated to the changes in BOD of water samples [84]. This technique used in BOD detection was demonstrated by Ahmad *et al.* [90] and Hur *et al.* [82]. A scheme of BOD biosensor is shown in Figure 8.

A BOD optical biosensor is generally designed with a respirometric and microbial type probe. Karube *et al.* [114] used *Pseudomonas putida*, and Riedel *et al.* [17] used *Trichosporon cutaneum* cells as the isolated bacterium for BOD detection. However, this probe has its limitations; oxygen consumption during the detection process will degrade the electrode. Electrochemical sensors based on the Clark-type oxygen electrode have been widely developed as the transducer of BOD sensors. Conceivably, the electrode used for oxygen measurement can be replaced by an optical (fluorescent) sensor. The major benefit of optical fibres over electrodes in the case of BOD measurements is that there is no oxygen consumption during measurement. Thus, no depletion of oxygen can occur [85] and it also does not require the frequent replacement of electrodes.

In addition, the direct method uses a fluorescent dye directly to measure the fluorescence quenching, where the reactive dye can react with specific compounds [62]. When used with an external fibre optic probe, the method takes advantage of ruthenium complexes. Ruthenium complexes are an attractive class of compounds for oxygen-stimulated fluorescence quenching in sensing applications. This is due to their high photochemical stability, high extinction coefficients, and absorption spectra the matches with the emission spectrum, for example, blue LED [115]. As a result, this fluorophore was immobilized in a photopolymerized hydrogel made from poly(ethylene glycol) diacrylate (PEGDA), [116] and was investigated for luminescence based tests suing fibre optic oxygen sensors [87].

**Figure 9.**
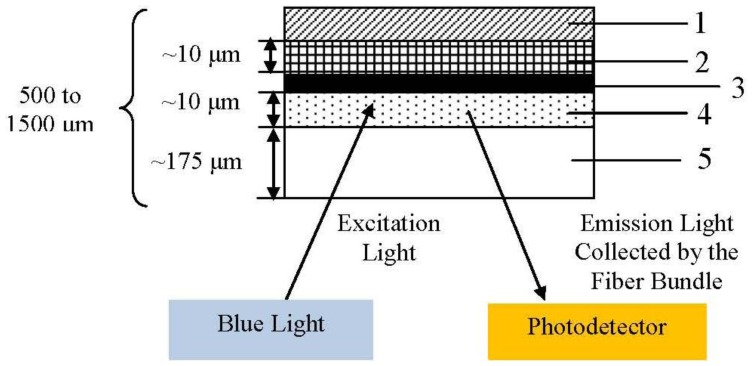
Cross-section of sensing film for BOD determination. Layer 1 is polycarbonate cover; Layer 2 is yeast immobilized in PVA; Layer 3 is charcoal acting as an optical isolator; Layer 4 is oxygen sensitive fluorescent layer; Layer 5 is inert and gas to impermeable polyester support (adapted from [85]).

A microbial system should offer low selectivity, high assimilability and high bio-oxidation activity for a broad range of organic compounds [114]. Due to the fact each microbial species has its metabolic deficiencies, the universality of BOD sensor is restricted. As a result, it is essential to select an appropriate microorganism for a biosensor [117]. The first fibre optic microbial sensor for determination of BOD was demonstrated by Wolfbels *et al.* [85] with a fluorescence-based technique. A simple description had been shown as Figure 9 below.

*Trichosporon cutaneum* cells immobilized in poly(vinyl alcohol) offer a biocompatible microenvironment for microorganisms and allow prompt response (diffusion) of organic compounds. Besides, tris(4,7-diphenyl-l,l-O-phenanthroline) ruthenium(II) perchlorate is used as the oxygen indicator. Excitation light (from the bottom) passes through the polyester support and excites fluorescence in the oxygen sensitive layer. Then, the emitted light is collected and guided by the fibre bundle to the photo detector. This research showed that estimation of BOD can be made rapidly, in about 5 to 10 min. It is possible to use this probe *in situ* for sewage plant effluents and municipal sewage monitoring, however, the short lifespan of the microbes and the thickness of cells are limitations that have to be overcome.

The strains were also studied by Karube *et al.* [86] using a conventional microorganism, *Pseudomonas putida*. This research demonstrates that there is a requirement for a maintenance-free microbial BOD sensor that has long term stability and is not affected by heavy metals. Nevertheless, the reproducibility of BOD sensors based on particular cells or activated sludge is often poor. Consequently, mixed strains on microorganisms immobilized within a single sensor were developed [117]. Kwok *et al.* [88] modified this sensor by immobilizing activated sludge *cum Bacillus subtilis* on oxygen sensing films and placing it underneath the sample vials. In an extensive study, Chen *et al.* [89] applied multiple microorganisms—*B.licheniformis*, *D. maris* and *M. marinus*—into a single microbial BOD biosensor.

## 8. Future Perspectives

Many researchers have studied the biochemical and genetically modified bacteria bio-luminescence. The microorganisms genetically modified to emit luminescence which are immobilized onto an exposed-core of a fibre optic, provide a convenient method to multiply the possibilities for rapid, simple and sensitive screening of the environmental conditions [118]. This application was presented by Belkin *et al.* [119] for pollution monitoring in soil and water. These discoveries have revolutionized the use of luminescent genes as fibre optics biosensors for environmental studies [120].

## 9. Challenges

In spite of the progress in the optical sensors, there are still some limitations that need to be addressed. For example, optical instruments are affected by susceptibility to surrounding variations, e.g., pH, turbidity, temperature, flow rate and so on. Optical fibres are susceptible to physical perturbations, such as bending, kinking and crushing. In addition, the assembly of optical components for the sensor also requires tremendously high precision and accuracy, especially for placement of optical components, which normally has a tolerance of less than a micrometer. This tolerance is vital to guarantee the reliability of the resulting device. This is where the real challenge lies in the case of FOS and control schemes [121]. Thus, fibre optics chemical sensors continue to attract strong research and development interest.

## 10. Conclusions

Some of the latest developments in colour, COD and BOD sensing using bulk optic and fibre optic approaches have been thoroughly reviewed. The design of FOS has evolved from the use of simple techniques based on intensity modulation to advanced techniques based on absorption and fluorescence analysis. In terms of applicability, although both the absorption and fluorescence-based sensors produce better performance, they are not widely used in the detection of COD and BOD. Perhaps this is due the large number of components in wastewater that cannot be differentiated by these methods alone. Further study to improve the effectiveness of these methods can be initiated especially on improving the selectivity, sensitivity, and robustness of FOS.

## Figures and Tables

**Table 1.** Wastewater pollution parameters: colour, COD, BOD and their conventional detection methods.

**Parameter**	**Method**	**Description (Off-Line)**	**Detection Range**	**Reference**
Colour	Visual comparison	Visually compared to evaluate the standard.		[9]
	Spectrophotometric	Determined from the light transmission characteristics.		[9]
Colour	Tri-stimulus filter	A limited number of wideband spectral energy readings are taken along the visible spectrum.		[9,10]
	ADMI tri-stimulus Filter Method (3 wavelength (WL) and 31 WL ADMI	An extension of tri-stimulus method. It is based on the use of the Adams-Nickerson chromatic value formula.	100 to 10,000 ADMI	[11]
COD	Open Reflux Method	Water sample oxidized with excess amount of Cr_2_O_2_^−7^ for 2 hours in strong acidic condition with heat supplied to determine residual Cr_2_O_2_^−7^ to quantify wastewater COD. Suitable for a wide range of wastes.	5 to 50 mg O_2_/L and exceed than 50 mg O_2_/L	[9]
	Closed Reflux, Titrimetric and Colorimetri Method	Refer to “Open Reflux Method”. This method is more economical in the use of metallic salt reagents.	40 and 400 mg/L	[9]
COD	Ultrasonic digestion	Sample digestion using sonication.	100, 200 and 610 mg/L. 5 to 20% less than the reference.	[12]
	Microwave digestion	Sample digestion using microwave.	77.8 to 776.5 ± 5.9 mg O_2_/L	[13]
	Energy to saving heating	Utilises electromagnetic induction heating.	4 to 200 mg/L ± 3.5%	[14]
	Photolysis	Using hydrogen peroxide or luminal for oxidizing organic compound.	100 to 1,300 mg/L	[8,15]
	Respirometric	Needs 5 to 7 days incubation in the dark.	Up to 300 ± 2 mg/L	[16]
BOD	Microbial sensor	Using isolated bacterium in polyvinyl alcohol.	2 to 200 mg/L	[17,18]
BOD	Potentiometric stripping analysis	Oxidation of species previously deposited on an electrode.		[19]
	Incubation in acidified permanganate	Reducing the incubation period to 20 min at 60 °C	7 to 330 mg/L	[20]

**Table 2.** The basic optical sensor topologies are built up with light source, fibre, and detector.

**Item**	**Types**	**Key Words**	**Reference**
Optical Fibre	Single mode fibre	(a) Core diameter: 9 μm (b) Cladding diameter: 125 μm	[33]
	Multimode fibre	(a) Step index fibres which core is uniform refractive index medium (i) Core diameter: 50 to 85 μm (ii) Cladding diameter: 125 μm (b) Gradient index fibres which core transverse refractive index variation nearly parabolic. (i) Core diameter: 50 to 85 μm (ii) Cladding diameter: 125 μm (c) It can couple large amount of light and is easy to handle both arising from its large core size.	[33]
	Silica cavity fibre	(a) A resonant optical cavity is allows a beam of light to circulate in a closed path.	[34]
	Hollow cavity fibre	(a) Structure comprises skin layer, porous support layer and fibre bore.	[35]
	Plastic fibres	(a) Fibres made from polymer materials such as PMMA. (i) Core diameter: ∼1 mm (ii) Cladding diameter: μm	[33]
Light Source	Light emitting diodes	(a) Low coherence length, broad spectral width, low sensitivity to back reflect light and high reliability.	[36]
	Laser diodes	(a) Exhibit high coherence, narrow line width and high optical output power, and more expensive.	[36]
Light Source	Super-radiant diodes	a) Operating properties between LED and LD and exhibit high power, low coherence device.	[36]
	He-Ne laser	a) Relatively low cost and ease of operation compared to other visible lasers producing beams of similar quality in terms of spatial coherence.	[33]
Detectors	Semiconductor photodiodes	a) Good for visible and near IR wavelengths. b) There is no bandwidth limitation due to the detector as such.	[33]
	Avalanche photodiodes	a) Sense low light levels due to the inherent gain because of avalanche multiplication, but need large supply voltage (100 V).	[33]

**Table 3.** Losses in silica glass due to presence of 1 ppm of different metals and OH^−^ ions as impurities.

**Impurities**	**Loss due to Impurity (dB/km)**	**Reference**
Ni^2+^	0.10	[33]
Fe^3+^	0.15	[75]
Cu^2+^	1.10	[75]
Cr^3+^	1.60	[75]
OH^−^ (water)	4.00	[33]

**Table 4.** Typical wavelength dependence of attenuation for a silica fibre.

**Wavelength (nm)**	**Loss due to impurity (dB/km)**	**Reference**
850	1.70	[75]
1,300	0.35	[33]
1,550	0.15	[75]
1,625	0.21	[33]

**Table 5.** Overview of the optical and fibre optic sensing for Color, COD and BOD detection.

**Parameter**	**Material**	**Optical Range (nm)**	**Detection Range (mg/L)**	**Sensing Method**	**Response Time**	**Advantageous/Limitation**	**Application**	**Ref.**
Color	Direct determination	514.5	1000	Optical (Absorption-Based)	5 min	Sample handling	Dyed fibres	[76]
Color	Leather Dye Mixture Color (Red-Blue-Yellow)	400 to 900	Differentiate mixture of color, errors5.6 to10.7 and 6.9 to12.5%	Optical (Absorption-Based)	Once the PLSR method is optimized, new samples can be determined.	Sample handling	Leather dye detection	[77]
Color	Indigo Solution	635	800 to 12000, errors 0.5%	Optical (Absorption-Based)	15 min	Real time monitoring	Denim yarn	[78]
Color	Red, Amber, Blue solution	300 to 900	36 to1000, errors 1%	FOS	10 spectra were taken and averaged	Sample handling	Textile industry-dye bath	[79]
COD	Direct determination	254 & 356	0 to360 ± 1.8	Optical (Absorption-Based)	∼2 min	Sample handling	Printing and dyeing wastewater	[80]
COD	Direct determination	excitation wavelengths from 250 to 600	13 to 456 ± 6%	Optical (Synchronous Fluorescence)	Standard laboratory device.	Sample handling	Waste water from urban and non-urban area	[3]
COD	Phthalatehydrogen potassium for oxidation	near-infrared (NIR) transmission & ultraviolet absorbance (254)	5 to 400 ± 2%	Optical (Absorption-Based)	---	Real time monitoring	Organic pollutant	[24]
COD	Potassium hydrogen phthalate solution	200 to 720	30 to 1000 ± 1%	Optical	5 min	Real time monitoring	Lakes, river or waste water	[81]
COD	Direct determination	UV-220 & 254 emission spectra 300 to 550	1.6 to 20.6 ± 3%	Optical	--	Real time monitoring	Urban river water	[82]
COD	Direct determination	258 to 380& UV	0 to 350 ± 6%	FOS	62 s	Real time monitoring	Wastewater quality monitoring	[83]
BOD	Direct determination	220 to 1,100; excitation/emission 280/350	0 to 400	Optical (Absorption and Fluorescence Technique)	Standard laboratory device.	Sample handling	Sewage sample	[84]
BOD	Direct determination	190 to 900	100 to 10,000	Optical	Standard laboratory device.	Sample handling	Dirty water, slurry water	[25]
BOD	Direct determination	Excitation 250 to 600 nm	5.2 to 208 ± 8%	Optical (Synchronous Fluorescence)	Standard laboratory device.	Sample handling	Waste water from urban and non-urban area	[3]
BOD	Direct Determination	220 to 1,100 ;excitation λ 250 to 400 nm; emission λ 300 to 550 nm	0.5 to 25.4 ± 3%	Optical (Absorption and Flurescence)	Standard laboratory device	Sample handling	Urban river water	[82]
BOD	Direct determination	254 nm and fluorescence intensity 270 ∼ 300, 310 ∼ 370, 370 ∼ 400 & 400 ∼ 530	Waste water = 6.5 to 139.9 ± 10%; River water = 1.3 to 1.9 ± 22%	Optical (Absorption and Synchronous Flurescence)	Predict BOD by using multiple regression analysis	Sample handling	River water samples wastewater treatment plant effluent	[28]
BOD	Tris(4,7-diphenyl-l,lOphenanthroline) ruthenium(II) perchlorate; *Trichosporon cufaneum*	480, excitation 610 nm.	3 to 110 ± 4%	FOS	3–10 min	Rapid feedback signal, very low costs	Sewage plant effluent and municipal sewage	[85]
BOD	*Pseudomonas putida*	Used current	1 to 10 ± 20%	FOS (Fluorescenc-Based)	15 min	Long-term stability, no calibration drift occurs, not affected by heavy metal ions and chlorine concentration	River Water	[86]
BOD	Ru(I1) polypyridyl complex	Red-excitation, Blue-emmision	--	FOS	--	Real time monitoring	Waste water	[87]
BOD	Tris(4,7-diphenyl-1, 10-phenanthroline) ruthenium(II) dye;*B. subtilis*, &activated sludge	Four LED blue light (460 nm)	25 to 60 ± 14%	FOS (Fluorescenc-Based)	15–30 min (by batch)	Sample handling	GGA, domestic and synthetic WW, OECD	[88]
BOD	4,7-diphenyl-1,10-phenanthroline Ru(dpp)_3_^2+^; *B. licheniformis, D. maris*and *M.marinus*	Blue LED (465 nm)	0.2 to 40	FOS (Fluorescenc-Based)	3.2 min	without dramatically affected by sodium chloride	Seawater	[89]

## Nomenclature

COD: Chemical Oxygen Demand
BOD: Biological Oxygen Demand
TOC: Total Organic Carbon
SS: Suspended Solid
DOC: Dissolved Organic Carbon
FOS: Fibre Optics Sensor
POF: Plastic Optics Sensor
n_1_ or n_core_: Refractive Index Core Fibre
n_2_ or n_clad_: Refractive Index Cladding Fibre
n_2_ material: Refractive Index Modified Cladding Fibre
I_o_: Incident Intensity
I: Transmitted Intensity
*α*: Attenuation Coefficient
L: Length of Fibre
K: Quantum Efficiency
*ε*: Molar Absorptivity
I: Intensity of the Incident Light
C: Concentration of Fluoresces
TIR: Total Internal Reflection
*d*_p_: Penetration Depth
*λ*: Wavelength of the Propagating Signal in the Optical Fibre
*θ*: Angle of Incidence Normal at the Interface
EW: Evanescent Wave
DOE: Department of Environmental, Malaysia
FBG: Fibre Bragg Grating
LPG: Long Period Grating
PVA: polyvinyl alcohol
PEGDA: poly(ethylene glycol) diacrylate
